# Heptose Sounds the Alarm: Innate Sensing of a Bacterial Sugar Stimulates Immunity

**DOI:** 10.1371/journal.ppat.1005807

**Published:** 2016-09-22

**Authors:** Ryan G. Gaudet, Scott D. Gray-Owen

**Affiliations:** Department of Molecular Genetics, University of Toronto, Toronto, Ontario, Canada; Nanyang Technological University, SINGAPORE

## PAMPs Are Indicators of Microbial Identity and Virulence

The first line of defense against infection, the innate immune system, identifies and responds to microbial threats. Central to this response is the discrimination of self from non-self. Pattern recognition receptors (PRRs) expressed by mammalian cells detect molecular signatures unique to microbes yet absent from the host. These molecules, termed pathogen-associated molecular patterns (PAMPs), are invariant structures broadly represented among microbial taxa and have essential roles in microbial physiology. Consequently, only an extremely select group of molecules have been found to function as PAMPs.

It is becoming increasingly clear that rather than being redundant, PAMPs are interpreted by the innate immune system as a “pathogenic barcode” that informs the host to both the identity and infectious risk posed by a microorganism. The combination of PAMPs presented, the tissue type affected, and the subcellular compartment contaminated represent just some of the contextual signals used by the host to identify and assess the microbial threat. For example, PAMP contamination of the host cytosol is interpreted as an indicator of microbial virulence since direct cellular invasion or virulence factor-mediated translocation is a prerequisite for their presence in this normally sterile site [1]. Such distinctions are critical to maintaining immune homeostasis since, despite their name, PAMPs are not unique to pathogens; instead, they can be found in virtually all microorganisms, including the commensal microflora that constitutively reside on body surfaces.

## Heptose Is a Sugar Unique to Gram-Negative Bacteria

**Fig 1:**
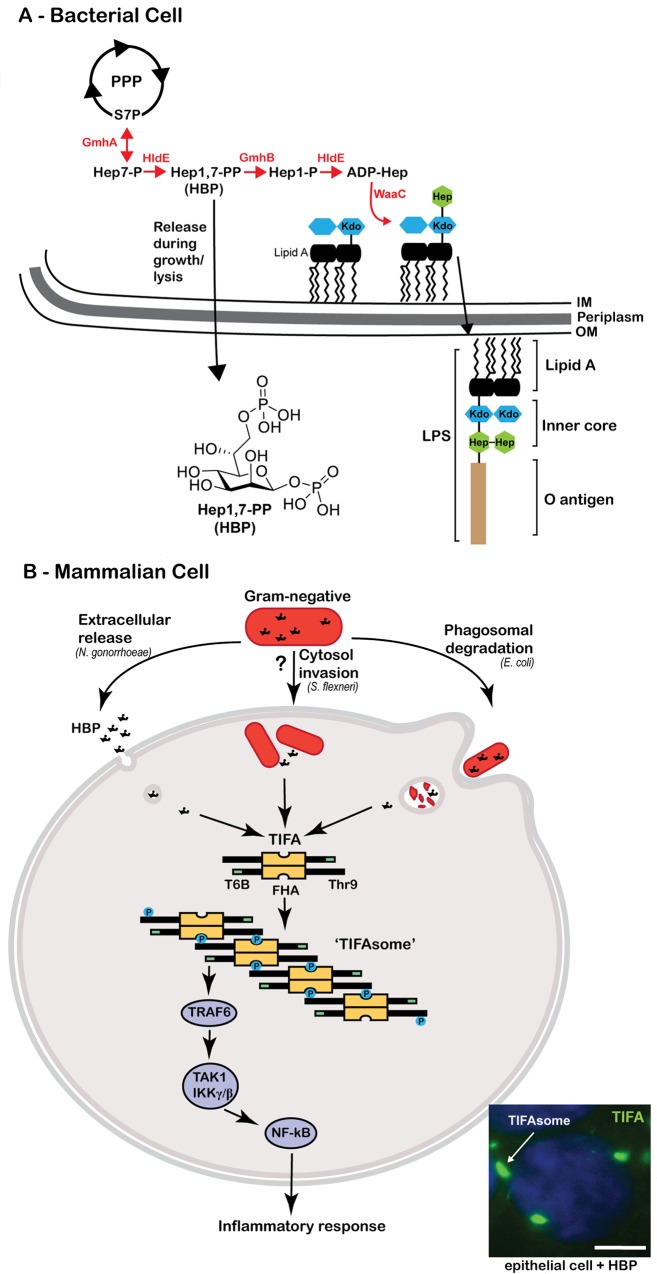
Host recognition of the bacterial metabolite HBP. (**A**) Gram-negative bacteria possess a unique branch from the pentose phosphate pathway (PPP) that generates ADP- heptose precursors (ADP-hep) for incorporation of heptose (green hexagon) into the inner core of the LPS structure. Hep-1,7-PP (HBP) is synthesized in the second step of this pathway and is released from bacteria during growth or lysis. (**B**) Summary of the manners by which gram-negative bacteria can present HBP to mammalian cells and activate the TIFA-signaling pathway. TIFA is an intrinsic anti-parallel dimer, possessing a Threonine residue (Thr9), a central forkhead domain (FHA), and a C-terminal TRAF6 binding site (T6BP; green rectangle). Upon HBP detection, Thr9 is phosphorylated, driving head-to tail oligomerization via intermolecular pThr9-FHA interactions. TIFA oligomers or the ‘TIFAsome’ recruits and activates TRAF6, driving canonical NF-κB activation and inflammatory signaling. Inset fluorescence micrograph depicts HBP-induced TIFAsome formation (green, white arrow) in human colonic epithelial cells treated with HBP for 2 hours. Nucleus is stained with DAPI (blue), scale bar 5 μm.

A central tenant to pattern recognition theory is that the structures the host has evolved to interpret as foreign are found solely in the microbial world. Gram-negative bacteria, for example, are defined by the presence of a second membrane outside of the cell wall, which serves as protection against both the environment and attacks by the host. The external leaflet of the outer membrane is composed of an amphipathic molecule termed lipopolysaccharide (LPS) (Fig 1A), which typically elicits an intense innate immune response. The hydrophobic base of LPS, termed lipid A or endotoxin, is a glucosamine-containing phospholipid acylated with a variable number of fatty acid chains [2]. This lipid A component is the innate immune agonist, since it activates the cellular PRRs toll-like receptor (TLR) 4 on the cell surface and the caspase-4/11 inflammasome within the cytoplasm [2,3]. Attached to lipid A are a series of repeated hydrophilic sugars, which can be subdivided into the core oligosaccharide and a longer repeating polysaccharide unit termed the O-specific chain [4]. The “inner core” region is typically composed of one to three residues of the seven carbon monosaccharide L-*glycero*-D-*manno-*heptose, which are often decorated with phosphate, pyrophosphate, or diphosphoethanolamine residues and serve to cross-link LPS molecules through divalent cations [5]. Notably, heptose is strictly derived from microbes, since gram-negative bacteria possess a unique five-step biosynthetic pathway that converts sedoheptulose-7 phosphate (S7P) from the pentose phosphate pathway (PPP) into ADP-heptose, the precursor for the heptose residues found in the inner core (Fig 1A) [6]. This biosynthetic pathway is well conserved across nearly all gram-negative phylogeny, with a few exceptions, including *Moraxella*, *Rhizobium*, *Francisella*, and *Legionella*, but is not found in other organisms [7].

## Heptose-1,7-bisphosphate (HBP) Is Gram-Negative Bacterial PAMP

The restriction of the ADP-heptose biosynthetic pathway to gram-negative bacteria implicates its metabolic constituents as possible signal(s) to the innate immune system. In fact, we recently discovered that the second molecule generated in this pathway, heptose-1,7-bisphosphate (HBP), elicits an inflammatory response from mammalian cells [8]. This metabolite is sensed within the host cytosol, inducing a robust inflammatory response by activating the transcription factor NF-κB. We reconstituted the ADP-heptose biosynthetic pathway in a cell-free system using recombinant enzymes to prove that HBP was both necessary and sufficient to induce inflammation. Curiously, though the end goal of this pathway is to synthesize LPS, the pro-inflammatory effect of HBP is not reproduced by heptose when incorporated into the intact LPS glycan structure.

There is strong selective pressure to synthesize ADP-heptose, as disruptions in this pathway generate bacteria with LPS truncations, referred to as “deep-rough mutants,” that can survive in the laboratory but are extremely susceptible to environmental stress [7]. In terms of virulence, deep-rough mutants are typically serum sensitive and avirulent in animal models. Indeed, in vitro and in vivo studies have revealed that deep-rough mutants of *Escherichia coli*, *Salmonella enterica*, *Shigella flexneri*, *Burkholderia*, *Neisseria meningitidis*, and *Neisseria gonorrhoeae* are avirulent [9–14]. Considering its microbial origin, the selective pressure for its generation, and the conservation of its synthesis pathway across gram-negative phyla, HBP satisfies the three defining characteristics of a PAMP [15].

## HBP Is Generated by a Wide Variety of Gram-Negative Bacteria but Signals in Different Cellular Contexts

A systematic analysis of the bacterial glycome indicated that heptose is among the most common monosaccharides restricted to the bacterial kingdom [16]. Moreover, the cellular abundance of LPS ensures the ADP-heptose pathway is highly active during bacterial growth and proliferation [2]. As such, HBP represents an intuitively satisfying PAMP. Indeed, we showed that immunoactive HBP is present in the cytosol of a wide variety of gram-negative bacteria [8]. However, the context in which HBP signaling is triggered differs considerably among bacterial species. For prototypical gram-negative bacteria such as *E*. *coli*, we found that HBP must be liberated from within the bacterial cytosol to activate host cells. We demonstrated that this can occur during extracellular bacteriolysis, or after opsonized bacteria are engulfed and degraded within the phagolysosome of macrophages [8]. Alternatively, we observed that the closely related pathogens *Neisseria gonorrhoeae* and *Neisseria meningitidis* each release abundant HBP during growth such that it accumulates in culture media [17]. Once liberated, extracellular HBP gains access to the host cytoplasm through dynamin-dependent endocytosis [8]. It is unclear if there exists an active mechanism of HBP export in *Neisseria* that is unique among gram-negative bacteria or if HBP release is instead indirect, attributable to some facet of the neisserial growth pattern yet to be recognized. Moreover, the requirement for cytosolic sensing makes it tempting to speculate that HBP might also function to alert the host to the presence of intracellular gram-negative bacteria. Both of these questions represent fascinating topics for future investigation.

## HBP Is Detected by a TIFA-Dependent Signaling Cascade

Germ-line encoded PRRs located on cellular membranes or in the cytosol sense PAMPs and trigger the production of proinflammatory cytokines. Following stimulation, each receptor recruits a defined set of downstream adaptor proteins through shared signaling domains, assembling into large multiprotein signaling complexes that in turn activate a common set of signaling mediators, including the TRAF family of proteins. TRAF6 specifically is an E3 ubiquitin ligase essential for signaling downstream of many PRRs [18].

Since host sensing of HBP occurred independently of any known PRR, we used a genome-wide RNA interference screen to uncover a novel cytoplasmic surveillance pathway that specifically detects HBP [8]. This revealed that HBP-driven inflammation was mediated by TRAF-interacting forkhead-associated protein A (TIFA), a ubiquitously expressed vertebrate protein with no clearly defined physiological function. Initially identified in a series of two-hybrid screens as a TRAF2- and TRAF6-binding protein, TIFA was annotated as a putative NF-κB activating protein when overexpression was found to activate NF-κB [19,20]. While TIFA normally exists as an antiparallel homodimer, overexpressed TIFA can become phosphorylated on threonine 9 (Thr9), leading to oligomerization via intermolecular binding of pThr9 of one dimer with the phospho-threonine binding forkhead domain of a different dimer [21]. Once formed, these oligomers are sufficient to activate oligomerization and polyubiquitination of TRAF6 in a cell free system [22]. The recently solved structure of TIFA supports a model in which “head-to-tail binding” between different TIFA dimers promotes oligomerization while leaving the C-terminal TRAF6 binding site exposed, creating a signaling scaffold that drives TRAF6 recruitment, oligomerization, and propagation of downstream signaling [23]. Despite this mechanistic insight, a role for TIFA in a cellular response remained elusive.

It was not until we reported that TIFA was essential for the cellular response to HBP that the function of TIFA became clear [8]. Whereas endogenous levels of TIFA are not phosphorylated, HBP induces the specific phosphorylation of TIFA on Thr9, leading to the pThr9-FHA-dependent intermolecular oligomerization events described above (Fig 1B). Whereas the overexpression studies had suggested TIFA was constitutively associated with TRAF6, we observed that HBP was required to promote the physical association of TIFA and TRAF6 in a more physiological context. In fact, HBP promotes the assembly of large TIFA and TRAF6-containing “TIFAsomes” that are evident by fluorescence microscopy (Fig 1B). Critically, TIFA is dispensable for macrophage responses to a variety of other PAMPs of bacterial, viral, or fungal origin, suggesting that its role is restricted to the HBP response. Whether TIFA is the receptor for HBP or a proximal signaling adaptor remains unknown. However, it is now clear that TIFA is a linchpin linking cytosolic detection of HBP with the common innate immune signaling hub TRAF6.

## Concluding Remarks

A major challenge of the future will be to define how innate sensing pathways integrate to distinguish pathogenic from commensal microorganisms. Understanding the full breadth of innate immune sensors, their microbial targets, and the context in which they are engaged is essential to achieving this goal. In this respect, HBP serves a flexible indicator of a diverse collection of bacterial species and lifestyles. It can function as an indicator of pathogenicity by signaling the presence of bacterial proliferation; alternatively, it can be interpreted as an indicator of bacterial death, since bacterial lysis or phagolysosomal degradation within macrophages may also liberate HBP (Fig 1B). Access to the cytosol is the limiting factor that determines whether HBP engages the TIFA signaling axis, and the amount of freely accessible HBP likely determines the immunological interpretation. Specifically, extracellular release may allow low level alert that gram-negative bacteria are in the tissues, bacteriolysis within the phagosome would allow a pulse alarm as HBP is released into the cytoplasm, while HBP generation during intracellular bacterial proliferation could permit accumulation and concentration in the cytosol creating an increasingly urgent signal. In this respect, the dose-dependent nature of HBP signaling that differs notably from the “all-or-nothing” response of inflammasome activating stimuli like cytosolic LPS [24] permits HBP to serve as a unique rheostat for bacterial proliferation.
